# The sambiloto (*Andrographis paniculata*) phytochemical as a feed additive for the prevention of *Eimeria* sp. infection in broilers

**DOI:** 10.5455/javar.2026.m1035

**Published:** 2026-03-24

**Authors:** Agustina Dwi Wijayanti, Wahyurafida Putri, Guntari Titik Mulyani, Dwi Priyowidodo, Ika Nindya Irianti

**Affiliations:** 1 Department of Pharmacology, Faculty of Veterinary Medicine, Universitas Gadjah Mada, Yogyakarta, Indonesia; 2 Sain Veteriner Graduate Program, Faculty of Veterinary Medicine, Universitas Gadjah Mada, Yogyakarta, Indonesia; 3 Department of Internal Medicine, Faculty of Veterinary Medicine, Universitas Gadjah Mada, Yogyakarta, Indonesia; 4 Department of Parasitology, Faculty of Veterinary Medicine, Universitas Gadjah Mada, Yogyakarta, Indonesia

**Keywords:** Anticoccidial, broiler, feed additive, performance, sambiloto

## Abstract

**Objectives:** This study aims to investigate the effectiveness of feeds containing sambiloto extracted in water (SWE) or ethanol (SEE) in improving the performance of broilers infected with *Eimeria* sp.

**Material and Methods:** A total of 80 ROSS 308 strain day-old chicks were divided into 4 groups: groups FSWE and FSEE (chickens infected with *Eimeria* sp. and fed with basal feed containing SWE and SEE, respectively), group F^+^ (chickens infected and fed basal feed), and group F^–^ (chickens not infected and fed basal feed). Infection was performed on day 14 by oral administration at a dose of 3×10^4^ oocysts for every chick. Body weight gain, feed conversion ratio (FCR), and survival rate were calculated during the treatment period. Anticoccidial efficacy variables were measured based on the oocysts per gram (OPG), oocyst ratio, oocyst value, and lesion scoring. Data were analyzed using one-way ANOVA and the Kruskal-Wallis test.

**Results:** The performance study results indicated that the highest body weight was achieved by the FSWE, and an ideal FCR was obtained from the FSEE, respectively. The lowest oocyst count was obtained in FSWE (*p* < 0.05), and the highest survival rate was observed in FSEE.

**Conclusions:** The study concluded that SWE and SEE have anticoccidial efficacy in broilers and offer benefits as feed additives in preventing *Eimeria* sp. infections.

## 1. Introduction

The ban on antibiotic use as an antibiotic growth promoter (AGP) is now worldwide. The increasing incidence of antimicrobial resistance (AMR) is attributed to the widespread use of antibiotics in livestock as growth promoters. Much research has been conducted on AGP substitutes by exploring various sources of compounds that have the effect of increasing body weight and maintaining health. *Andrographis paniculata* (mentioned as “sambiloto” by Indonesian people) is a plant known as the king of bitter because of its extremely bitter taste in every part of it. This plant grows abundantly in Asian countries, including India, Sri Lanka, Pakistan, Malaysia, and Indonesia. The plant exhibits a broad spectrum of pharmacological effects, some of which are highly beneficial, including hepatoprotective, antimicrobial, antifungal, antioxidant, anti-inflammatory, antipyretic, anticancer, and antidiarrheal properties [1]. The benefits of sambiloto reported by Ardika et al. [2] are anti-inflammatory and anti-infective agents because of the inhibition of lipopolysaccharide-induced tumor necrosis factor (*TNF)-α* and *caspase-3* expression. Andrographolide is an active compound that has several pharmacological effects, including anti-inflammatory, antibacterial, antiviral, antitumor, and immune-regulatory properties [3]. According to Jaidee et al. [4], the biological effectiveness of andrographolide remains stable in a relatively acidic environment, allowing for storage at low temperatures and requiring only short-term processing.

The phytogenic feed additive impacts growth performance and intestinal structure, potentially improving nutrient absorption and jejunal integrity in broilers [5]. By 2020, approximately 60% of global poultry producers had transitioned to antibiotic-free practices in response to rising consumer demand, and alternative feed additives, such as pre-probiotics, enzymes, organic acids, and phytogenic compounds, had become popular in poultry production [6]. As briefly mentioned in previous studies, the sambiloto phytochemical properties were hoped to inhibit the lesions in the gastrointestinal tract of broilers caused by coccidiosis.

Coccidiosis, an infection caused by *Eimeria* sp., negatively affects broiler chicken performance and causes economic and production losses [7]. Its annual cost is estimated to be about $11.93 billion due to a reduction in broiler performance, increased mortality, and the expense of medication and vaccines [8]. *Eimeria* is an intercellular intestinal protozoan parasite that causes an enteric disease that causes malabsorption, enteritis, depressed weight gain, uniformity issues, increased FCR, and mortality [9]. This study aimed to determine the effectiveness of sambiloto water extract (SWE) and sambiloto ethanolic extract (SEE) as feed additives in broilers infected with *Eimeria* sp. to maintain performance and inhibit the development of coccidiosis. Several studies have proven that herbs, such as Artemisia, clove, tea tree, and thyme, are effective in reducing coccidiosis by reducing the number of oocysts [10]. It is hoped that sambiloto extracts have potential as a phytogenic feed additive with beneficial effects, including anticoccidal properties, and that they can replace AGP in a more effective way to inhibit the rate of AMR.

## 2. Materials and Methods

### 2.1 Ethical approval

The methodology and experimental procedures involving animals in the study have been approved by the Ethical Committee of Research of the Faculty of Veterinary Medicine, Universitas Gadjah Mada, through letter No. 54/EC/FKH/int/2025.

### 2.2. The production of Eimeria sp. isolates

Isolation, purification, and production of *Eimeria* sp. oocysts were carried out based on the method of Shirley [11] with modifications. The domestic chicken diagnosed with coccidiosis and suffering from hemorrhagic diarrhea was obtained from local farming in the Sleman, Yogyakarta regency. The animal was euthanized by decapitation in the Islamic slaughter way, then necropsied, and the intestines were scraped to collect oocysts. The intestinal scraping was homogenized with Aquadest, then centrifuged at 2500 RPM for 5 min in conical tubes.

The centrifuge process is repeated twice, then saturated sugar solution is added to the conical tubes until the surface is convex. After 5 min, a glass object was placed over the tube to collect the oocysts and covered with a deck glass. The oocysts were harvested with a Pasteur pipette into a small conical tube and sporulated for 1–2 days. The sporulated oocysts were identified under the microscope (AmScope B120C 40X-2500X with Optilab camera^®^ Advance Lite).

### 2.3. Experimental design and treatment

Eighty of the ROSS 308 strain day-old chicks, basal feed, basal feed containing sambiloto water extract (SWE, water:sambiloto extract 1:1), and feed containing sambiloto ethanolic extract (SEE, ethanol:sambiloto extract 1.37:1) were obtained from producer PT Nugen Bioscience Indonesia. The extracts contained in the basal feed are 1% for both SWE and SEE. The day-old chicks were randomly divided into four groups (*n* = 20), with two groups receiving basal feed containing SWE (sugarcane waste extract) or SEE (sugarcane ethanol extract), while the other two groups received basal feed only. After 14 days, the groups that were fed SWE, SEE, and one with basal feeds were infected with *Eimeria* sp. at 3 × 10^4^ oocysts per chick by oral administration. The groups were then named FSWE and FSEE (chickens infected with *Eimeria* sp. and fed with SWE and SEE, respectively), group F^+^ (chickens infected and fed basal feed without extracts), and group F^–^ (chickens not infected and fed with basal feed). The F^–^ group (negative control group) was caged at a distance from the 3 infected groups to prevent contamination. All types of feed and drinking water were provided *ad libitum* for 35 days. The cage used in this study was an infectious litter cage of the open house type, located in the Animal Health Education and Training Unit, Faculty of Veterinary Medicine, Universitas Gadjah Mada.

### 2.4. Data collection

#### 2.4.1. The performances

Body weight gain was calculated weekly, while FCR and survival rates were measured at the end of treatment. The FCR value was obtained by dividing the feed consumption by body weight gain. The clinical signs were recorded daily to monitor the development of coccidia infection and the health status of the broilers.

#### 2.4.2. The anticoccidial effectiveness

The oocyst per gram (OPG) was obtained from the fresh feces of all groups. The oocysts were counted quantitatively in a counting room using the McMaster method [12]. Feces were homogenized with aqua dest and mixed with a magnetic stirrer. The solution was placed on double-object glass, supplemented with saturated sugar at a 1:1 (v/v) ratio, and left for 15 min before being counted under a microscope. The number of oocysts counted was multiplied by 50 to obtain the number of oocysts per gram of feces. From the oocyst amount, we then derived the oocyst ratio, which was the number of oocytes in the healthy group or experimental group divided by the number of oocytes in the infection group.


\[{\mathrm{Oocyst\;ratio\; = \;}}\frac{{{\mathrm{Number\;of\;oocytes\;in\;the\;healthy\;group\;or\;experimental\;group}}}}{{{\mathrm{Number\;of\;oocyst\;in\;infected\;group}}}}{\mathrm{\; \times \;100\% }}\]

The oocyst’s value is calculated based on the ratio of OPG, derived from the oocyst, to determine the infection rate of the treatment group [13]. The lesion score value was measured after a week of the broilers being infected, during which they were infected for 3 weeks (days 21, 28, and 35). This was done by euthanizing and necropsying 3 chickens from each group and then measuring the intestinal lesion scores based on Johnson’s method (Table 1).

**Table 1.** The intestinal scoring lesion score [14].

Score	Intestinal conditions
0	No lesion.
1	Few petechiae or bleeding points in the intestinal wall; no intestinal wall thickening; normal intestinal content.
2	More lesions were found; there is mild bleeding in the intestinal lumen, thickening of the intestinal wall, and normal intestinal content.
3	There is hemorrhage or a massive central core, severe thickening of the intestinal wall, and less content in the intestines.
4	There are ballooning intestines or a caseous core, severe bleeding, and no feces or intestinal contents; a dead chicken is given a score of 4.

### 2.5. Data analysis

The software used in the data analysis was Microsoft Excel and SPSS version 27. Some data were calculated semi-quantitatively with scoring and percentages. Data that were normally distributed and homogeneous were analyzed using one-way ANOVA. Meanwhile, the data that were not normally distributed or homogeneous were analyzed using the Kruskal-Wallis test. The significance was set at *p* < 0.05.

## 3. Results

### 3.1. Feed analysis and identification of Eimeria sp.

The ingredient analysis was conducted to determine the nutrient and mineral content of the feeds. Table 2 presents the results of the proximate analysis and mineral content of the feeds, which were analyzed by the Laboratory of Nutrition Biochemistry, Department of Nutrition and Feed, Faculty of Animal Husbandry, UGM.

**Table 2.** The proximate and mineral analysis results of feeds.

Feed type	Dry material (%)	Ingredient Percentage based on dry material				Calcium		Phosphorus	
		Ash	Crude protein	Crude fat	Crude fiber	%Ca	mg/kg	%PO_4_	mg/kg
Basal	90.17	6.04	17.78	7.21	3.89	0.437	4368.226	0.549	1789.908
FSWE	90.48	5.77	17.82	5.99	4.05	0.421	4211.637	0.524	1710.709
FSEE	89.94	5.85	16.58	6.62	3.57	0.448	4478.754	0.562	1834.912

Note: FSWE = basal feed containing 1% of sambiloto water extract; FSEE = basal feed containing 1% of sambiloto ethanolic extract.

**Figure 1.**
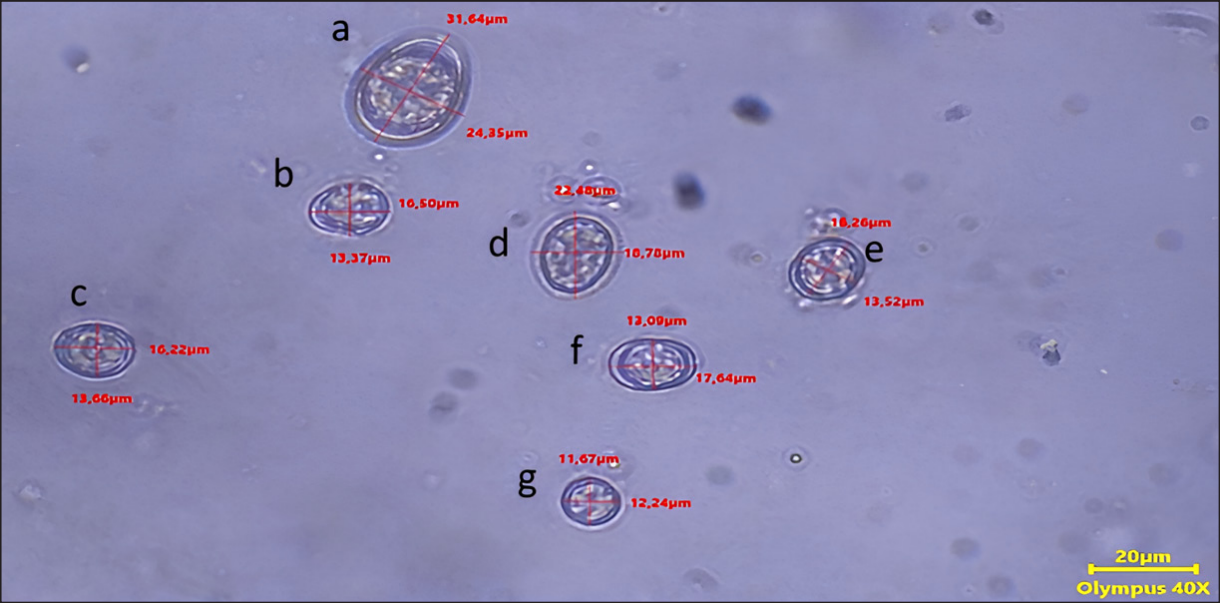
Identification of *Eimeria* sp. from infected groups: (a) *E. maxima*; (b, c, f) *E. necatrix*; (d) *E. tenella*; (g) *E. mitis*.

Figure 1 shows the species of *Eimeria* found in infected groups 5 days after being infected with sporulated oocysts. The identification of species was based on the morphology and diameter of the oocyst [9, 15]. In one field of view of the microscope at 40x magnification, four species of *Eimeria* sp. were identified: *E. maxima, E. necatrix, E. tenella*, and *E. mitis*. Other species found were *E. acervuline* and *E. brunetti*, as well.

### 3.2. Performance and anticoccidial effects

The clinical signs were observed post-infection every day in all groups. Clear disease symptoms were observed in the F^+^ group, including lethargy, dull feathers, and diarrhea in some chickens, with the highest mortality rate also being noted. The FSWE and FSEE groups exhibited no obvious disease symptoms, although chicken deaths continued to occur. The body weight gain (Table 3) shows that the effect of infection inhibits weight gain starting in week 3, indicated by the significance of weight gain between infected groups and the control group (F^–^). Meanwhile, Table 4 presents the feed conversion and survival rates of broilers following the treatments.

**Table 3.** The average body weight gain during treatments (gm).

Week	Body weight gain (gm)			
	FSWE	FSEE	F^+^	F^–^
1	162.20 ± 15.02^a^	165,80 ± 13.26^a^	161.07 ± 23.962^a^	156.40 ± 11.41^a^
2	440.01 ± 41.77^a^	458.47 ± 33.28^a^	462.73 ± 78.183^a^	440.93 ± 31.95^a^
3	712.50 ± 131.76^b^	664.18 ± 65.24^b^	689.73 ± 123.09^b^	880.27 ± 104.61^a^
4	1220.80 ± 352.87^ab^	1087.88 ± 240.70^b^	1145.50 ± 258.81^b^	1465.83 ± 145.45^a^
5	2070.11 ± 87.68^ab^	1741.40 ± 111.57^b^	1912.33 ± 121.26^ab^	2152.22 ± 238.50^a^

Note: The different superscripts in the same rows indicate significant differences (*p* < 0.05). FSWE = chickens infected with *Eimeria* sp. and fed with basal feed containing SWE, FSEE = chickens infected with *Eimeria* sp. and fed with basal feed containing SEE, F^+^ = chickens infected and fed with basal feed, and F^–^ = chickens not infected and fed with basal feed.

**Table 4.** The feed conversion ratio and survival rates of the broilers after the treatment.

Group	FCR (gm feed weight/gm gain weight)	Survival (%)
FSWE	2.10	83
FSEE	2.07	93
F^+^	2.17	66
F^–^	1.58	100

Note: FSWE = chickens infected with *Eimeria* sp. and fed with basal feed containing SWE, FSEE = chickens infected with *Eimeria* sp. and fed with basal feed containing SEE, F^+^ = chickens infected and fed with basal feed, and F^–^ = chickens not infected and fed with basal feed.

The OPG counted the number of fresh feces every day for six days, a week after the infection. The most severe infection was observed on day 21, characterized by a higher number of OPG in the infected groups (Table 5). On days 22–24, there was a relative decrease in the number of oocytes in the infected group, indicating self-limiting disease, which will be discussed in the discussion session.

**Table 5.** The oocyst amount calculation (OPG) from feces after infection.

Broiler’s age	Groups			
	FSWE	FSEE	F^+^	F^–^
Day 20	37,100.00 ± 13,283.35^b^	5,116.66 ± 4,132.29^a^	2,033.33 ± 378.59^a^	0 ± 0^a^
Day 21	221,350.00 ± 43,068.23^d^	139,550.00 ± 45,239.88^c^	50,383.33 ± 7,823.25^b^	0 ± 0^a^
Day 22	21,866.67 ± 6,711.99^b^	116,00.00 ± 6,732.19^c^	5,233.33 ± 4,346.93^a^	0 ± 0^a^
Day 23	5,550.00 ± 2,965.21^a^	36,183.33 ± 14,471.72^b^	3,350.00 ± 2,001.8^a^	0 ± 0^a^
Day 24	22,783.33 ± 12,241.56^b^	4,600.00 ± 1,769.18^a^	7,950.00 ± 2,835.48^ab^	0 ± 0^a^
Day 25	14,116.67 ± 9,848.39^ab^	17,066.67 ± 2,676.44^ab^	62,250.00 ± 40,200.00^b^	0 ± 0^a^

Note: The different superscripts in the same rows indicate significant differences (*p* < 0.05). FSWE = chickens infected with *Eimeria* sp. and fed with basal feed containing SWE; FSEE = chickens infected with *Eimeria* sp. and fed with basal feed containing SEE; F^+^ = chickens infected and fed with basal feed; and F^–^ = chickens not infected and fed with basal feed.

Based on the OPG, the oocyst ratio was counted, and the oocyst value was established as a conversion from the oocyst ratio (Tables 6, 7).

The lesion score was measured weekly (days 21, 28, and 35) after the day of infection. The necropsy results from each group were analyzed, and a lesion score was determined based on the Johnson & Reid method. The score was a set of conditions from the small intestine mucosa and the content of the lumen (Table 1), and the scoring results are presented in Table 8.

## 4. Discussion

The common feed additives tested in poultry include phytogenic feed additive groups, such as essential oils [16, 17], herbal extracts [18], organic acids [19], prebiotics and probiotics [20], and enzymes [21]. *Andrographis paniculata* extract has been studied by Hasanain et al. [22] as an additive in broiler feed and has been shown to improve the carcass quality. According to Irianti et al. [23], the sambiloto extract contains high andrographolide and tannin, which have anticoccidial properties against *E. tenella, E. maxima*, and *E. acervulina*. Freitas et al. [7] mention that *Eimeria* sp. infection reduces the average daily gain because the aggressive action of protozoa causes lesions in the gut epithelium, reducing the absorption of nutrients and energy. The infection also reduced the FCR and increased the mortality [8].

Table 3 shows the decrease of ADG in all infected groups compared to F^–^, as well as an increase in FCR values (Table 4). The mortality rates of FSWE and FSEE were lower compared to F^+^, indicating the presence of anticoccidial potency in sambiloto extract. Plant-based feed additives, including plant extracts, have been shown to exert diverse effects on non-infected chickens, potentially enhancing growth rates and optimizing feed conversion efficiency [24]. The reduced performance of the broilers was clearly caused by the pathogenic species of *Eimeria* identified after infection (Figure 1). The mixed infection caused by *Eimeria sp*. in this study was identified mostly as a pathogen species [25, 26].

The phytochemical properties of SWE and SEE, as reported in the published results of our preliminary study [23], contain abundant phenolic compounds, including gallic acid and tannin, which account for more than 7% and 8% of the total secondary metabolites, respectively. This study also reported that the andrographolide content in SEE (5.72%) is higher than in SWE (0.3%), but conversely, the flavonoid content in SWE is higher (1.72%) than in SEE (0.82%). According to Hadidi et al. [27], gallic acid is a potent antioxidant that exerts anti-inflammatory effects by inhibiting inflammatory cytokines and enzymes, making it a potential therapeutic agent for inflammatory diseases, such as coccidiosis in broilers. Tannic acid is a gastroprotective that regulates inflammation and oxidative stress [28] and has an effect as an antioxidant and anticancer, anti-allergic, and antimicrobial property [29].

*Eimeria* infections are self-limiting because the number of asexual, pathogenic cycles is genetically predetermined. Often, this process also stimulates a protective, species-specific immunity in the host, preventing future infections from the same *Eimeria* species [30]. As shown in Table 5, on day 21, the oocyst count of FSWE and FSEE was higher than that of F⁺, which may be due to the difference in the number of oocysts infected in the concentration or to the presence of certain species that grow rapidly. On days 22–24, it tends to decrease, which may reflect the self-limiting nature, and then increases again on day 25. Unavailability of OPG data at the end of treatment was a limitation of this study. However, the performance results and lesion scores measured over a longer study period revealed that the *Eimeria* infection was prevented. The anticoccidial property of FSWE and FSEE might inhibit the oocyst production, where the oocyst counts are lower than F⁺ in general. The ratio of oocysts, along with the oocyst value, is a term used in the quantification and analysis of parasitic oocysts, particularly in studies concerning parasitic diseases such as coccidiosis and malaria. The oocyst ratio helps researchers determine the effectiveness of a treatment or intervention [31]. Tables 6 and 7 present the calculations of both oocyst ratio (%) and oocyst value, comparing the infection rates of groups fed SWE and SEE with those of both the infected and non-infected groups.

**Table 6.** The conversion of the oocyst ratio to the oocyst value.

Oocyst ratio (%)	0–1	1–25	26–50	51–75	76–100
Oocyst value	0	5	10	20	40

**Table 7.** The rate of *Eimeria* sp. infection by the oocyst value.

Broiler’s age	Oocyst value			
	FSWE	FSEE	F^+^	F^–^
Day 20	40	30	40	0
Day 21	40	40	40	0
Day 22	40	40	40	0
Day 23	30	40	40	0
Day 24	40	20	40	0
Day 25	10	10	40	0

Note: FSWE = chickens infected with *Eimeria* sp. and fed with basal feed containing SWE; FSEE = chickens infected with *Eimeria* sp. and fed with basal feed containing SEE; F^+^ = chickens infected and fed with basal feed; and F^–^ = chickens not infected and fed with basal feed.

**Figure 2.**
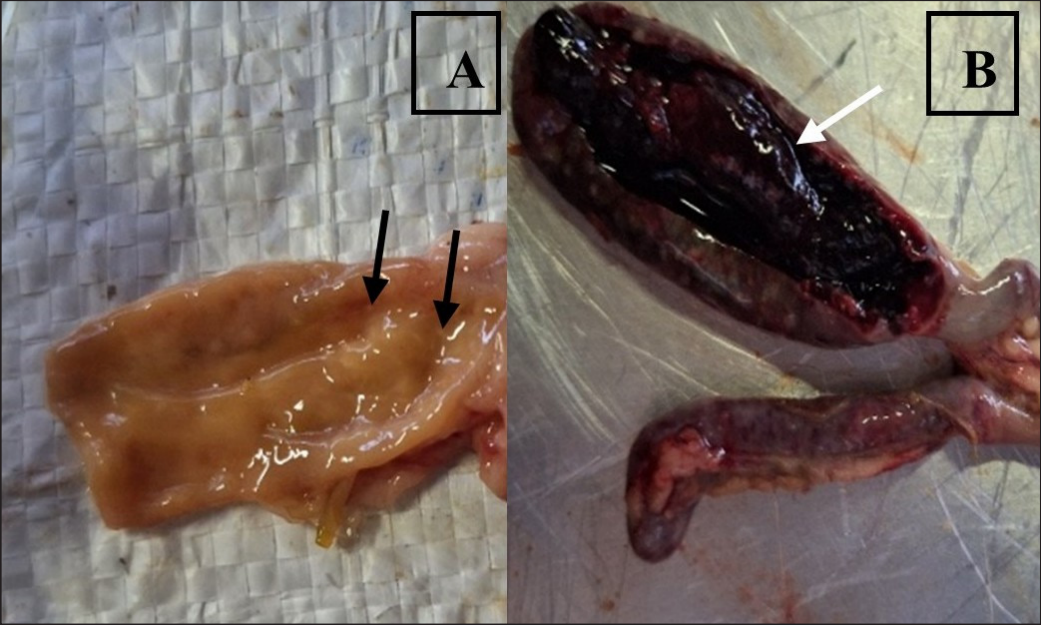
The macroscopic lesions of intestines caused by *Eimeria* sp. infection. A) White spot lesions on the duodenum (black arrows), B) Severe hemorrhage of the cecum (white arrow).

The scoring of intestinal lesions reflected damage caused by *Eimeria* sp. (Table 8) and was quantitatively measured using the Johnson method (Table 1) to assess the progress of healing throughout the treatment period. When coccidiosis challenge studies are conducted, a gross evaluation of the intestine and oocyst shedding is performed once at 6–7 days post-infection [32]. Figure 2 shows the macroscopic lesions of the intestines of infected groups on day 21, or 7 days after infection. The severe hemorrhages of the cecum and white spots of the duodenum typically result from *E. tenella* and *E. acervulina* infections, respectively [33]. In this study, sambiloto extracts, used as a feed additive, were able to reduce and heal intestinal lesions, as evidenced by the absence of lesions on day 35.

**Table 8.** The averages of the scoring results on days 21, 28, and 35, respectively.

Group	Lesion scoring		
	Day 21	Day 28	Day 35
FSWE	0.67 ± 1.15^a^	0 ± 0^a^	0 ± 0^a^
FSEE	0.67 ± 1.15^a^	0.33 ± 0.57^a^	0 ± 0^a^
P (+)	1.33 ± 0.57^a^	1.33 ± 0.57^a^	2.67 ± 0.57^b^
P (–)	0 ± 0^a^	0.67 ± 1.15^a^	0 ± 0^a^

Note: The different superscripts in the same rows indicate significant differences (*p* < 0.05). FSWE = chickens infected with *Eimeria* sp. and fed with basal feed containing SWE; FSEE = chickens infected with *Eimeria* sp. and fed with basal feed containing SEE; F^+^ = chickens infected and fed with basal feed; and F^–^ = chickens not infected and fed with basal feed.

According to Ahmad et al. [34], it will not be possible to maintain the current level of chicken production without a comprehensive anticoccidial management program. In Europe, almost all poultry farms use antiparasitic drugs as feed additives for pullets and broiler breeders for a period of 12 to 16 weeks, as well as for broiler chickens for nearly their whole lives. Somehow, the residues of coccidiostats and antimicrobial drugs in poultry products may pose a public health concern [35]. Owing to the serious risks associated with the use of coccidiostats, there is a robust feeling that synthetic drugs should be replaced by natural additives [36]. The anticoccidial effect resulting from FSWE and FSEE is the scientific basis for using sambiloto extract as a feed additive for broilers, particularly on farms with a high prevalence of coccidiosis.

## 5. Conclusions

Based on the results and discussion, it is concluded that sambiloto extract, when used as a feed additive in broilers, offers benefits in maintaining performance and reducing *Eimeria* sp. infection. The anticoccidial action of sambiloto extract in feed significantly repaired the intestinal damage caused by *Eimeria* sp. The phytochemicals contained in sambiloto have been proven beneficial as a feed additive and are likely to replace synthetic antimicrobial compounds, particularly in preventing coccidiosis in broilers.

## Data Availability

The data presented in this study are available from the corresponding author upon reasonable request.

## List of abbreviations

ADG, average daily gain; AGP, antibiotic growth promoter; AMR, antimicrobial resistance; ANOVA, analysis of variance; F^+^, chickens infected and fed with basal feed; F^–^, chickens not infected and fed with basal feed; FCR, feed conversion ratio; SEE, Sambiloto ethanolic extract; FSEE, chickens infected with *Eimeria* sp. and fed with basal feed containing SEE; FSWE, chickens infected with *Eimeria* sp. and fed with basal feed containing SWE, OPG, oocysts per gram; ROSS 308, a specific strain of day-old chicks; SPSS, Statistical Package for the Social Sciences; SWE, sambiloto water extract; TNF-α, tumor necrosis factor alpha; UGM, Universitas Gadjah Mada.
